# Identification and validation of a major quantitative trait locus for spike length and compactness in the wheat (*Triticum aestivum* L.) line Chuanyu12D7

**DOI:** 10.3389/fpls.2023.1186183

**Published:** 2023-07-04

**Authors:** Mingxiu Wang, Jing Lu, Rong Liu, Yunfang Li, Donghui Ao, Yu Wu, Lei Zhang

**Affiliations:** ^1^ Chengdu Institute of Biology, Chinese Academy of Sciences, Chengdu, China; ^2^ University of Chinese Academy of Sciences, Beijing, China; ^3^ Department of Agriculture, Forestry and Food Engineering of Yibin University, Yibin, China

**Keywords:** wheat, spike length, spike compactness, plant type, MAS

## Abstract

Spike length (SL) and spike compactness (SC) are crucial traits related to wheat (*Triticum aestivum* L.) yield potential. In this study, a backcrossed inbred lines (BILs) population segregating for SL/SC was developed by using a commercial variety chuanyu25 as recurrent parent and a backbone parent Chuanyu12D7. Bulked segregant analysis (BSA) combined with the Wheat 660K SNP array was performed to conduct quantitative trait locus (QTL) mapping. A major and stable SL/SC QTL (designated as *QSl/Sc.cib-2D.1*) was identified on chromosome 2DS, explaining 45.63-59.72% of the phenotypic variation. *QSl/Sc.cib-2D.1* was mapped to a 102.29-Kb interval by flanking SNPs AX-110276364 and AX-111593853 using a BC_4_F_2:3_ population. Since *QSl/Sc.cib-2D.1* is linked to the *Rht8* gene, their additive effects on plant type and spike type were analysed. Remarkably, the superior allele of *QSl/Sc.cib-2D.1* combined with *Rht8* can increase SL and TGW, and decrese SC without any apparent trade-offs in other yield-related traits. In addition, the closely linked kompetitive allele-specific PCR (KASP) markers of this locus were developed for marker-assisted selection (MAS) breeding. Four genes within the physical interval were considered as potential candidates based on expression patterns as well as orthologous gene functions. These results laid the foundation for map-based cloning of the gene(s) underlying *QSl/Sc.cib-2D.1 and* its potential application in wheat ideotype breeding.

## Introduction

Bread wheat (*Triticum aestivum* L.), with a broad adaptation, is the most widely planted crop in the world, with approximately 216 million hectares of the planting area in 2019 and the total global production of 760 million tons in 2020 (FAOSTAT Database, 2020, https://www.fao.org/faostat/en/#home). However, the current growth rate of wheat production cannot meet future demand (Saini et al., 2022). Previous studies have identified spike morphological traits that directly or indirectly contribute to wheat yield (Wurschum et al., 2018; Zhang et al., 2022). Among them, the spike length (SL) and spike compactness (SC) are of great significance for wheat spike development, plant architecture and wheat yield (Wu et al., 2012; Gao et al., 2015). Thus, the detection of quantitative trait loci (QTLs) involved in SL/SC could provide a genetic basis for high-yield wheat breeding.

As a sink organ, spike morphology can affect many yield-related traits, such as spikelet number per spike, grain number per spike (GNS) and thousand grain weight (TGW) (Kuang et al., 2020; Xu et al., 2022a; Zhang et al., 2022). Elucidating the genetic basis of spike morphology will pave the way for breeding source-sink balanced wheat varieties. Reports have shown that spike length is positively correlated with aboveground biomass, biomass per plant, harvest index and yield (Donmez et al., 2001; Faris et al., 2014; Li et al., 2016). In addition, increasing spike length can lead to a decrease in spike compactness (Zhai et al., 2016), which may increase the resistance of wheat to fusarium head blight (Buerstmayr et al., 2009). However, studies have also found that increasing spike length does not affect spike compactness, thereby increasing yield (Cui et al., 2011). The spike length trait is controlled by multiple genes, is weakly influenced by the environment, and has a high broad-sense heritability (Cui et al., 2011). To date, an increasing number of QTLs associated with SL/SC have been identified over the last decades (Jantasuriyarat et al., 2004; Johnson et al., 2008; Cui et al., 2011; Faris et al., 2014; Wu et al., 2014; Deng et al., 2017; Shi et al., 2017; Guo et al., 2018; Liu et al., 2018; Xu et al., 2018; Chai et al., 2019; You et al., 2021). However, few QTL related to SL/SC had been fully studied, which hampered the usage of their favorable allele in wheat breeding.

There are three well-known genes affecting spike morphological traits: *Q*, *Compactum* (*C*), and *Sphaerococcum* (*S*). The *Q* gene encoding a transcription factor of the AP2 (APETALA 2) family located on 5A chromosome (Simons et al., 2006), resulting in increased spike compactness. In the history of wheat cultivation, the *Q* gene has not only changed the seed threshability and spike compactness, but also increased the planting area of wheat (Faris and Gill, 2002; Simons et al., 2006; Xie et al., 2018; Xu et al., 2018). The *C* locus located on 2D contributes to increased spike compactness (Johnson et al., 2008). The *S1* gene, located on the 3D chromosome, influences a number of traits such as spike compactness, hemispherical glume and spherical grain (Salina et al., 2000). In addition, three sets of genes, *photoperiod* (*Ppd*) (Boden et al., 2015), *vernalization* (*Vrn*) (Wang et al., 2014) and *earliness per se* (*Eps*) (Faricelli et al., 2016), are also involved in spike development. Moreover, many QTLs contributing to spike morphological traits have been identified and only a few of these have been cloned. For example, the cloned *TaCol-B5*, encoding a CONSTANS-like protein, increases both the spikelet number per spike and SL, as well as the number of tillers, leading to an increase in field-based grain yield (Zhang et al., 2022). The disclosure of *TaCol-B5*, with a potential yield increase of ~12%, is a milestone for understanding the molecular mechanisms of spike morphological and yield-related traits.

Previous studies have shown that the average spike length of super-high-yielding varieties is longer than that of general high-yield varieties, while the spike compactness is lower than that of general high-yield varieties (He et al., 2002). The varieties with low spike compactness generally have large and neat grains and high 1000-grain weight, which is conducive to high yield. Modification of plant architecture and spike morphology (such as spike length and compactness) is a key driver of wheat yield potential but is challenging because of the negative correlation between different factors. Since the 1960 s, the utilization of dwarf/semi-dwarf varieties (with alleles of *Rht* loci) in wheat breeding has significantly reduced plant height and improved lodging resistance and wheat productivity (Flintham et al., 1997). However, most of the dwarfing genes showed negative effects on grain yield or other agronomic traits. For example, it was reported that *Rht4 on* 2BL reduced plant height by 17%, but had a negative correlation with grain weight (Gooding et al., 2012). The dwarfing gene *Rht5* on 3BS can significantly delay heading date and increase the number of fertile tillers, but reduce GNS, grain yield and harvest index (Rebetzke et al., 2012; Daoura Goudia et al., 2014). These negative effects limit the application of *Rht5* in wheat breeding. To date, only a small number of *Rht* genes have been applied in wheat breeding. For example, the *Rht8* near-isogenic line (NIL) with semi-dwarf stature decreases SL and increases SC but displayed a 10% yield penalty (Kowalski et al., 2016). Therefore, when utilizing reduced height genes in breeding programs, attention should be paid to the coordination of spike morphological traits, source-sink correlation, growth rhythm, light-temperature response and other factors, and the optimization and assembly of excellent genes. Accordingly, the yield-increasing potential under the background of excellent genotypes can be transformed into the actual productivity of wheat varieties.

In the present study, a BC_1_F_7_ (backcross inbred lines, BILs) population was constructed with CY25 as recurrent parent for mapping the major QTL for SL/SC. Using bulk segregant analysis (BSA) with the Wheat 660K Array strategy, closely linked markers for major QTL were developed and used for validation and fine mapping in a BC_4_F_2:3_ population. The additive effects of the major QTL and its closely linked gene *Rht8* were analyzed. The ideotype of wheat was then discussed in terms of plant type and spike type. Additionally, candidate genes for the QTL were predicted.

## Materials and methods

### Plant materials

In this study, two hundreds and sixty-two BC_1_F_7_ lines (backcross inbred lines, BILs) derived from the cross of Chuanyu25 and Chuanyu12D7 were constructed for genetic analysis and QTL mapping. The recurrent parent is CY25, and the donor parent is Chuanyu12D7. Chuanyu25 (CY25, with a pedigree of Jinding-1//Yumai35/Lang9247) was an excellent awnless wheat variety (carrying *Yr9* and *Yr17*, and semi-dwarf gene *Rht-D1b*) with moderate to high resistance to stripe rust, high resistance to powdery mildew and moderate resistance to scab. It is a high-yield cultivar wildly cultivated in Sichuan Province, bred by Chengdu Institute of Biology (CIB), Chinese Academy of Sciences in 2015 (Certification *No.* 2015012). Chuanyu12D7 with a pedigree of G214//Yumai35/Lang9247 was an improved line of Zhongzhi3586, which introduced as a resistance resource of stripe rust from the Institute of Plant Protection, Chinese Academy of Agriculture Sciences in the 1980s. It has many excellent agronomic traits including suitable plant height, good general combining ability, floriferousness and fruitfulness, and high heritability of long spike. Over the past decade, Chuanyu12D7 has therefore been widely used in wheat breeding in Sichuan Province of China. The spike length of Chuanyu12D7 is significantly longer than that of CY25, but with a similar spike number per spike, suggesting that this combination is suitable for major QTL identification of SL. In addition, an advanced BC_4_F_2:3_ population with 122 progenies (CY25/4/CY25/3/CY25//CY25/Chuanyu12D7 F_2:3_) was created for QTL fine mapping.

### Field trials

The BILs population was evaluated alternately at the Shifang Agroecosystem Experimental Station (SF, 104°11′E, 31°6′N, and elevation 521 meters, located in the northern Chengdu Plain) and the Barkham Agroecosystem Experimental Station, the Chinese Academy of Science (BH, 102 °6′E, 31°55′N, and elevation 2670 meters, located in the east of the Qinghai-Tibet Plateau) in Sichuan Province, China.

Each trial used a randomized block design with single row single seed sowing. The row length was 1.5 m, row spacing was 0.3 m, each row was sown with 12–15 seeds, each plant in a row spaced 0.15 m apart. For all the field trials, the sowing density was 15 seeds per row, with a distance of 15 cm between the plants within a row. Seeds were hand sown at the end of October in SF and plants were harvested in the middle of May of the next year at physiological maturity. Harvested seeds planted at the beginning of June in BH, and plants were harvested at the end of September. To be specific, the BC_1_F_2_ was planted in SF during 2018-2019, BC_1_F_2:3_ in BH of 2019, BC_1_F_4_ in SF during 2019–2020, BC_1_F_5_ in BH of 2020, BC_1_F_6_ in SF during 2020–2021, BC_1_F_7_ in SF during 2021–2022. Uniform disease and pest management practices were implemented in the field experiments to ensure accurate assessment of phenotype.

### Phenotypic evaluation and statistical analysis

For all populations in each year, three representative plants with similar growth status were selected to investigate the following traits: plant height (PH), spike length (SL), total spikelet number per spike (TSS), sterile spikelet number per spike (SSS), fertile spikelet number per spike (FSS) and grain number per spike (GNS) were determined from the main spike of the three plants, and productive tiller number (PTN). PTN was the number of tillers that could produce a spike. PH was measured from the base of the plant to the top of the main spike excluding awns. SL of the main spike of each plant was measured as the length from the base of the rachis to the terminal spikelet excluding awns. Spike compactness (SC) is equal to TSS divided by SL. After harvesting the whole plants, the main spike of each plant was manually threshed and GNS was calculated. Thousand grain weight (TGW), grain length (GL) and grain width (GW) were evaluated by weighing three samples using SC-G software (Wseen Co., Ltd, China). The mean values of these traits in each line were used for QTL mapping. The phenotypic data of BC_1_F_4_, BC_1_F_5_, BC_1_F_6_ and BC_1_F_7_ were used in this study. The phenotypic data of BC_4_F_2:3_ population planted in 2021–2022 Shifang and 2022 BH were also collected.

Descriptive statistics, Student’s *t*-tests, normal distributions, bivariate two-tailed Pearson’s correlation coefficients and analysis of variance were calculated using the Microsoft Office 365 Excel and IBM SPSS Statistics 26 (IBM Corp., Armonk, NY). The normality of the data obtained was tested using the Shapiro-Wilk test (*P* < 0.05).

### Bulk segregant analysis with wheat 660 K SNP array

To identify the major QTL controlling SL/SC in BILs, bulk segregant analysis (BSA) combined with the Axiom^®^ Wheat 660K Genotyping Array was performed. In the BILs population, 50 extreme lines with stable long spike length and 50 lines with short SL were selected to construct mixing pools. Five lines were selected from each of the two parents. Leaf tissue samples from all selected lines were collected separately and frozen in liquid nitrogen for genomic DNA extraction using the CTAB method (Sharp et al., 1988) and then mixed into four pools. Each single sample was mixed to have a total mass of 2 μg and a concentration greater than 300 ng/μl. The mixed pool with long SL was called the long bulk (L-B), and the one with short SL was called the short bulk (S-B). DNA quality and concentration were measured by agarose gel electrophoresis and Thermo Scientific NanoDrop 2000 spectrophotometer. Genotyping of two parents and two pools was performed using the Axiom^®^ Wheat 660K SNP Array by Beijing CapitalBio Technology Co., Ltd. (https://www.capitalbiotech.com/). Single nucleotide polymorphisms (SNPs) with call rate (CR) > 94 and dish QC > 0.82 were selected as PolyHighResolution SNPs. The SNPs that were monomorphic, heterozygous genotype (e.g., C/G, T/A) and of poor-quality with more than 10% missing values were excluded from further analysis. The homozygous SNPs (e.g., G/G, A/A, T/T, C/C), with high-quality genotyping properties, were retained for further analysis. By comparing the SNPs in two mixed pools and two parents, the polymorphic SNPs were obtained. Subsequently, the distribution of SNPs on each chromosome was represented in a 1 Mb window. The physical interval most likely to contain SL loci was then determined. The physical positions of all SNP markers were searched in IWGSC RefSeq v1.1 (The International Wheat Genome Sequencing Consortium (IWGSC), 2018).

### Development of molecular markers

The polymorphic SNPs in the targeted region were converted into Kompetitive Allele Specific PCR (KASP) markers based on the wheat 660 K SNP analysis. Moreover, eighteen SNPs in the target region were extracted from the WheatOmics platform (Ma et al., 2021) and converted to KASP markers. All the KASP markers were designed on the Galaxy platform (Galaxy, 2022). The specificity of the KASP markers was first tested in two parental lines and then in the BILs population. The primers were designed carrying FAM (5′ -GAAGGTGACCAAGTTCATGCT-3′) or HEX (5′ -GAAGGTCGGAGTCAACGGATT-3′) tail at the 5′ terminal. The procedure of KASP assays was followed by the protocol of LGC Genomics or LGC Biosearch Technologies (Teddington, Middlesex, UK) (http://www.lgcgroup.com/). The KASP assays were tested in a 96-well plate, with 10 μl reaction volume containing 5 μl of 2× KASP master mix, 0.8 μl of SNP primer mix, 4–50 ng of DNA template with the addition of ddH_2_O up to 10 μl. Touchdown PCR cycling was performed as follows: hot start activation at 95°C for 10 min, followed by 10 touchdown cycles (95°C for 20 s, touchdown 61°C, drop 0.6°C per cycle for 40s), and then 30 cycles of amplification at 95°C for 20 s, and 55°C for 40 s. The fluorescence signal was detected at 35°C for 30 s using QuantStudio™ Real-Time PCR System (Thermo Fisher Scientific).

Twelve simple sequence repeat (SSR) makers in the target chromosomal region were also selected from the public domain (http://wheat.pw.usda.gov) for mapping, of which three SSR markers were tested as polymorphic between two parents. The diagnostic gene marker of *Rht8* was used in this study to select *Rht8* alleles and one SSR marker near to *Rht8* was also collected (Chai et al., 2022). Moreover, the sequence between the two flanking markers was extracted using the Jrowse of the WheatOmics platform (Ma et al., 2021). SSRs were found based on the extracted sequence using MISA-web (Beier et al., 2017). PrimerServer of the WheatOmics platform was used to design genome specific SSR markers. PCR amplification for SSR primers was performed using 12.5 μl 2× GS Taq PCR Mix for PAGE (Genesand Biotech Co., Ltd, China), 2 μl primer mix, 1 μl template DNA (approximately 100 ng/μl) and ddH_2_O to a final reaction volume of 25 μl. The PCR amplification as follows: 94°C for 5min; 30 cycles of 94°C denaturation for 20s, 50-60°C primer annealing for 20 s, and 72°C extension for 25 s; and final extension at 72°C for 5min. The 8% non-denaturing polyacrylamide gels (PAGE) was used for separating PCR products.

### QTL mapping and verification

All polymorphic KASP and SSR markers were used to construct the genetic map using the MAP module in QTL IciMapping 4.2 (Li et al., 2007). In the study, the following steps were taken to identify and analyze QTLs: Firstly, the “bin” function in IciMapping 4.2 was employed to eliminate redundant markers based on their segregation patterns in the mapping population. The parameters “Missing Rates” and “Distortion Value” were set at 20% and 0.01, respectively. Secondly, QTL detection was conducted using inclusive composite interval mapping for Additive QTL (ICIM-ADD) based on the biparental populations (BIP) function in QTL IciMapping 4.2. The P1BC1RIL function was used as the QTL analysis model. Lastly, the bin markers were ordered using the Kosambi mapping function (Kosambi, 1943), and a logarithm of odds (LOD) of 3.0 was set by performing 1,000 permutation tests with a scanning walking step of 1.0 cM. The analysis excluded any missing phenotypes. The study computed the phenotypic variances explained (*PVE*) by QTL and estimated additive effects (Add) in different environments. QTLs that were consistently identified in at least three environments and in combined analysis with a phenotypic variation explained of ≥10% were regarded as major QTLs. Genetic linkage maps were generated using both QTL IciMapping 4.2 and MapChart V2.32 software (Voorrips, 2002). The physical positions of all SNP probes were obtained by blasting against the newest genome assembly of IWGSC RefSeq v2.1 (http://202.194.139.32/blast/blast.html).

To validate the major QTL, flanking markers of the major QTL were used to genotype the BC_4_F_2:3_ population. These new SNPs and SSR markers were used to screen the BC_4_F_2:3_ population. Three-Point Analysis Mechanism was applied by using the MAPMAKER/EXP Version 3.0b (Lander et al., 1987). Kosambi’s mapping function was used to determine the order of the markers (Kosambi, 1943). Moreover, the additive effects of QTLs in SL, SC and other yield-related traits were evaluated using Student’s *t*-test. The corresponding physical distances were determined by blasting sequences of flanking markers to the IWGSC RefSeq v2.1 (Zhu et al., 2021).

The major QTL was named according to the rules of International Rules of Genetic Nomenclature (https://wheat.pw.usda.gov/ggpages/wgc/98/Intro.htm). Specifically, the QTL was named beginning with “Q” followed by the trait name abbreviation, research department and the chromosome. The abbreviation “cib” was used to refer to the Chengdu Institute of Biology.

### Detection of *Rht8*

The diagnostic marker for *Rht8* gene, a cleaved amplified polymorphic sequence (CAPS) marker, was used to genotype the population which was developed based on the CG–T sequence substitution in RNHL-D1 (Chai et al., 2022). The marker included two pairs of primers: *Rht8-2F* and *Rht8-2R for* the first PCR cloning, and *Rht8-6F* and *Rht8-R2* for the second PCR cloning. After amplification, we digested the 586-bp PCR products using BsmI (New England Biolabs, cat. no. R0134L) at 65°C for 4 h and then separated them in a 2% agarose gel to determine the sizes of digested PCR products. The wild-type genotype with *rht8* showed three bands with the 585-bp band from original PCR, and 424 bp and 162 bp bands from the BsmI digestion. In contrast, the semi-dwarf plants with *Rht8* showed only a single band of 585 bp from the original PCR (**Supplementary Figure 1**). This CAPS marker used to genotype all lines in populations.

### Prediction of candidate genes

The physical positions of the flanking markers for the major QTL were obtained by blasting against IWGSC RefSeq v1.0 (Appels et al., 2018), RefSeq v2.1 and *Ae. tauschii* Aet v5.0 (Wang et al., 2021). Candidate genes between the flanking markers were obtained with functional annotations by using the IntervalTool of the WheatOmics platform (Ma et al., 2021). Expression pattern analysis of these genes was performed by using expVIP (http://www.wheat-expression.com/cite). Orthologous gene analysis was conducted by using the HomologFinder tool of the WheatOmics. Meanwhile, non-synonymous SNPs associated with candidate genes were collected using the Wheat 660 K results and SNP information from the WheatOmics.

## Results

### Phenotypic variation and correlation analysis

**Figure 1:**
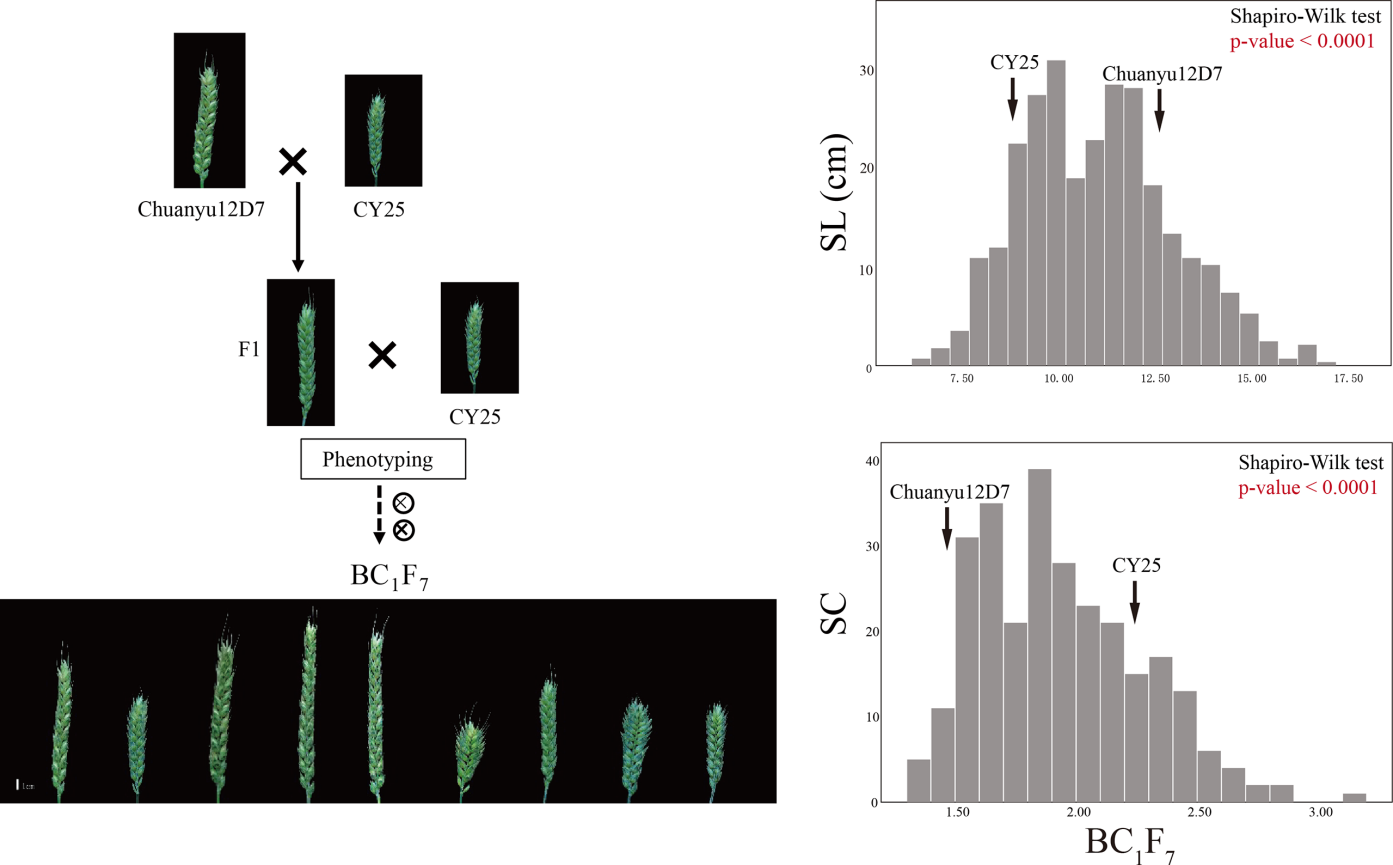
The construction of the BIL population and frequency distribution of the BC_1_F_7_ for spike length (SL) and spike compactness (SC). Scale bar = 1 cm.

Significant differences for SL and SC between CY25 and Chuanyu12D7 were detected in all environments (**Table 1**). As observed, CY25 had a shorter and more compact spike than Chuanyu12D7, with a wide variation in the progeny (**Supplementary Figure 2**). In the BILs population, SL and SC exhibited wild and significant variations in all environments, with coefficient of variation (CV) ranging from 12.96 to 21.98% and 13.04 to 22.56%, respectively (**Table 1**). The SL, SC, GNS, TGW and PH of two parental lines showed significant differences. On the contrary, TSS, PTN, FSS, SSS, GW and GL between two parental lines showed no significant difference at the 0.01 significance level (**Supplementary Table 1**). In BC_1_F_4_, BC_1_F_5_, BC_1_F_6 and_ BC_1_F_7_ populations, the frequency distribution for SL/SC displayed an obvious deviation from the normal distribution, suggesting major genes underlying the variation of SL/SC in the BILs population (**Figure 1**; **Supplementary Figure 3**). Moreover, the frequency distribution for PH also displayed a deviation from the normal distribution (**Supplementary Figure 4**).

**Table 1:** Summary statistics of spike length (SL) and spike compactness (SC) of the parental lines and the BIL population in different environments.

Traits	Env	Parents		The BILs population				
		CY25	Chuanyu12D7	Mean	Range	Kurtosis	Skewness	CV%
SL (cm)	BC_1_F_4_	9.53^***^	13.4	11.46 ± 1.85	5.5-15.8	-0.52	-0.07	0.16
	BC_1_F_5_	6.07^***^	9.64	7.09 ± 1.31	4.5-11.5	-0.48	0.47	0.18
	BC_1_F_6_	8.72^***^	11.12	9.97 ± 1.67	5.5-16	-0.7	0.15	0.17
	BC_1_F_7_	9.37^***^	12.63	11.05 ± 1.8	6.5-17.1	-0.15	0.4	0.17
SC	BC_1_F_4_	2.31^***^	1.67	1.96 ± 0.33	1.36-3.45	0.88	0.74	0.17
	BC_1_F_5_	2.63^**^	2.1	2.66 ± 0.45	1.67-4.17	-0.5	0.28	0.17
	BC_1_F_6_	2.12^***^	1.69	1.95 ± 0.35	1.15-3.88	1.24	0.74	0.18
	BC_1_F_7_	2.35^***^	1.5	2.01 ± 0.34	1.31-3.52	0.24	0.62	0.17

Env, environments; CV, coefficient of variation.

^***^ indicate the significant level at P < 0.001.

**Figure 2:**
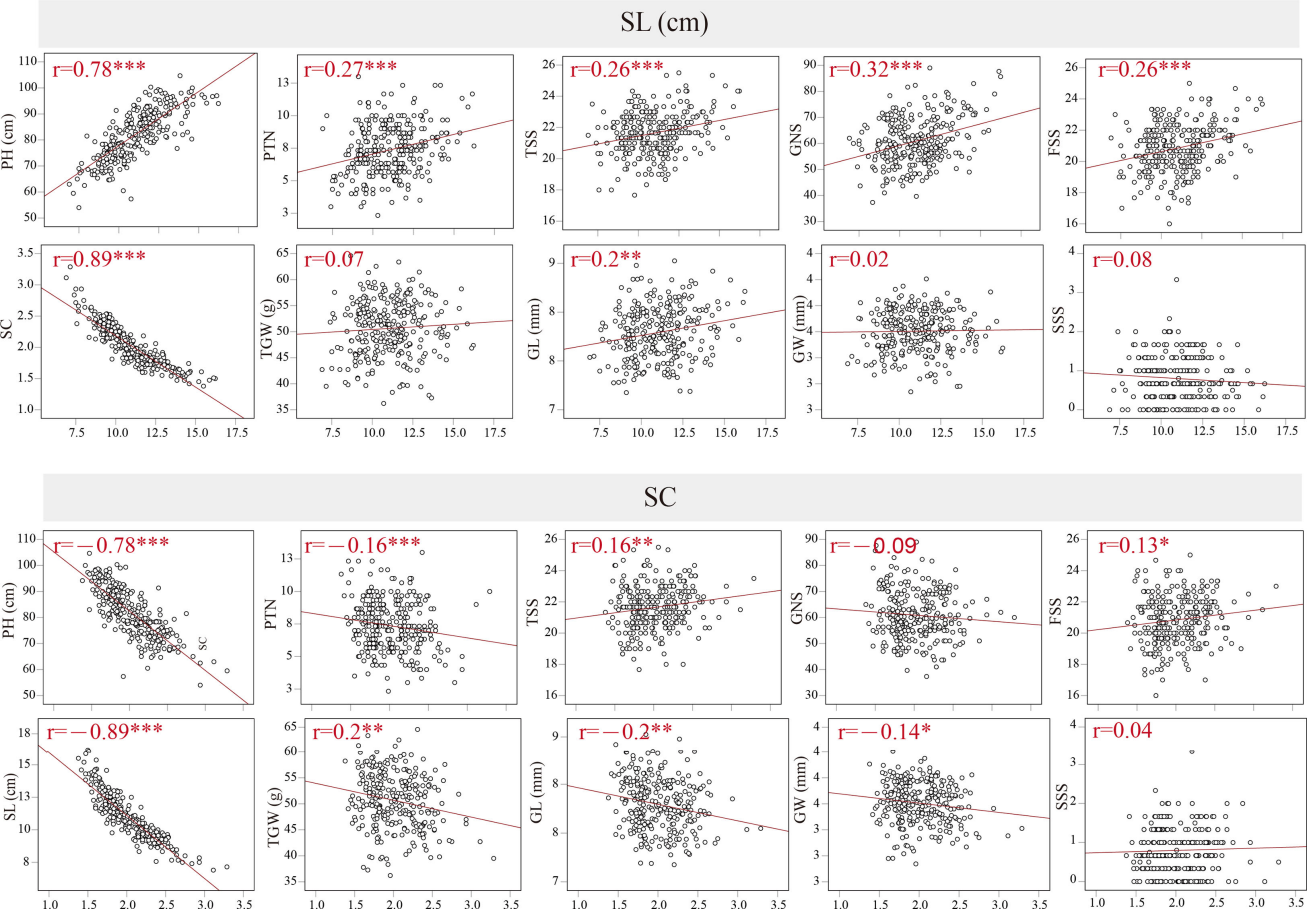
Coefficients of pairwise Pearson’s correlations between spike compactness (SC), spike length (SL), plant height (PH), productive tiller number (PTN), total spike number per spike (TSS), grain number per spike (GNS), fertile spikelet number per spike (FSS), thousand-grain weight (TGW), grain width (GW), grain length (GL), sterile spikelet number per spike (SSS) in the BILs population. ^*^, ^**^ and ^***^ significance at *P* < 0.05, *P* < 0.01 and *P* < 0.001, respectively.

The coefficients of pairwise Pearson’s correlations between SC, SL and other yield-related traits were determined to assess trait correlations using the average data from BC_1_F_4_–BC_1_F_7_ (**Figure 2**). In the BILs population, SL was significantly and negatively correlated with SC, with *r* = -0.89 and *P* < 0.001. SL displayed strongly positive correlations with PH, PTN, TSS, GNS, FSS and GL. In contrast, SC displayed significantly negative correlations with PH, PTN, TSS, FSS and GL (*P* < 0.01), and a weak negative correlation with GW (*P* < 0.05). In addition, SC was positively correlated with FSS (*P* < 0.05) and TGW (*P* < 0.01). Between SL, SC and SSS, however, no significant correlation was observed. Furthermore, the coefficients of pairwise Pearson’s correlations for spike length and spike compactness in different environments were evaluated in the BIL population, with the Pearson’s correlation value of 0.63–0.8 and 0.68–0.72 for SL and SC, respectively (**Supplementary Table 2**).

### BSA and wheat 660 K analysis

The BSA combined with the Wheat 660K SNP Array was performed to detect the genomic regions for SL and SC, and the results were compared with the IWGSC RefSeq v1.1 genome assembly. All Dish QC (DQC) values of the four bulks are greater than 0.98, demonstrating the high quality of the test results (**Supplementary Table 3**).

**Figure 3:**
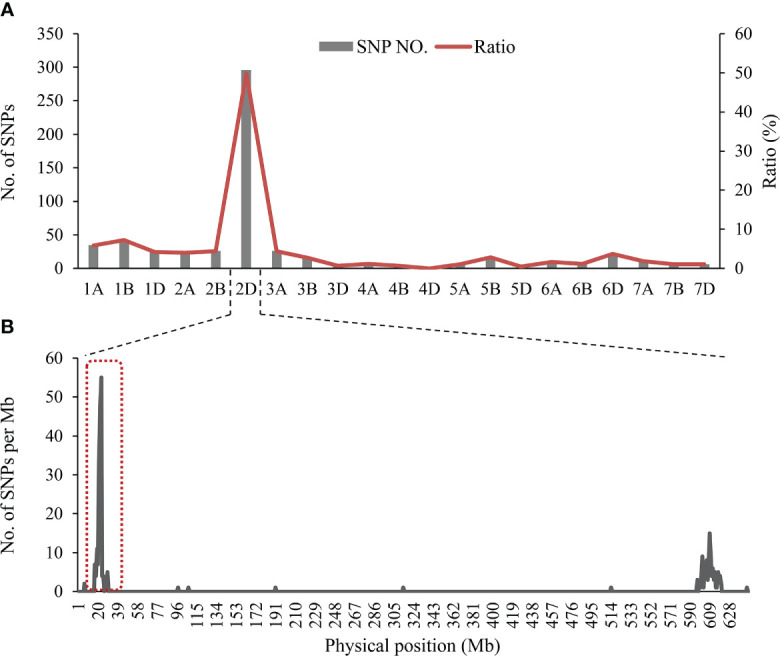
**(A)** Distribution of polymorphic SNPs on each chromosome, transverse axis for 21 wheat chromosomes, longitudinal axis for the number of SNPs and the proportion of SNPs to the total SNPs, **(B)** The number of SNPs on chromosome 2D based on position in the IWGSC RefSeq v1.1.

After filtering and clustering, a total of 608 homozygous polymorphic SNPs between the two parental pools and two mixed pools were identified, and 298 of these SNPs (49.01%) were distributed on chromosome 2D (excluding 16 unknown anchoring SNPs) (**Supplementary Table 4**). Meanwhile, the number of SNPs in a 1-Mb interval on 2D was counted. Most of the polymorphic SNPs on the short arm of chromosome 2D (2DS) were significantly enriched in the physical interval of 22-26 Mb in the Chinese Spring RefSeq v1.1 (**Figure 3**). From these results, it was tentatively determined that a locus controlling SL is most likely to be located on chromosome 2DS.

### QTL mapping for spike length and spike compactness

To locate QTL associated with SL and SC, sixty chromosome-specific SNPs on 2DS in the target region chosen for conversion to KASP markers were screened; 20 out of 60 SNP markers were successful in discriminating the two parents (**Supplementary Table 5**). To saturate the genetic map, 36 SSR markers were developed, one of which exhibited polymorphism between two parents. Five SSRs from online database, one new SSR marker (*CIB6*) in this region were also used, resulting in a total of 26 markers to genotyping the entire BC_1_F_7_ population for constructing the genetic map (**Supplementary Table 6**). The genetic map of the target region on 2DS spanned 36.89 cM in length.

**Figure 4:**
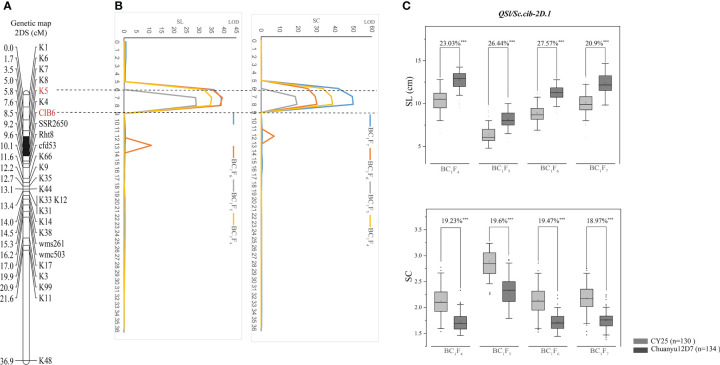
The genetic map and LOD value of the *QSl/Sc.cib-2D.1* **(A, B)** and its effects on spike length (SL) and spike compactness (SC) in the BILs population (**C**). The flanking markers are shown in red and the black segment represents the interval of *QSl/Sc.cib-2D.1*. ^***^ represents significance at *P* < 0.001.

Using the phenotypic data of SL evaluated in BC_1_F_4_, BC_1_F_5_, BC_1_F_6_ and BC_1_F_7_, a major QTL, *QSl.cib-2D.1*, was identified and located in a 2.66 cM interval by flanking markers *CIB6* and *K5*, explaining 45.63-57.87% of the phenotypic variance with LOD values ranging from 28.97-38.99 (**Table 2**). For SC, *QSc.cib-2D.1* was stably detected in all four generations by flanking markers *CIB6* and *K5*, accounting for 47.52-59.72% of the phenotypic variation with LOD values ranging from 19.44-49.92. *QSl.cib-2D.1* and *QSc.cib-2D.1* were located in the same interval, suggesting pleiotropism of this QTL. To simplify, the genomic region encompassing SL and SC was hereafter referred to as *QSl/Sc.cib-2D.1* (**Figure 4**). Allele of Chuanyu12D7 for *QSl/Sc.cib-2D.1* contributed to longer spike length (with additive effect from 0.65 to 1.26) and less compact spike (with additive effect from -0.23 to -0.17).

**Table 2:** Quantitative trait loci (QTL) for spike length (SL) and spike compactness (SC) in different environments.

Trait	QTL	Env	Position (cM)	Left marker	Right marker	LOD	PVE (%)	Add
SL	***QSl.cib-2D.1***	BC_1_F_7_	5.84-8.5	*K5*	*CIB6*	38.99	51.84	1.26
		BC_1_F_6_	5.84-8.5	*K5*	*CIB6*	39.37	54.67	0.91
		BC_1_F_5_	5.84-8.5	*K5*	*CIB6*	28.97	57.87	0.65
		BC_1_F_4_	5.84-8.5	*K5*	*CIB6*	35.11	45.63	1.25
	*QSl.cib-2D.2*	BC_1_F_6_	12.72-13.13	*K35*	*K44*	11.04	11.56	0.42
SC	***QSc.cib-2D.1***	BC_1_F_7_	5.84-8.5	*K5*	*CIB6*	49.92	59.72	-0.23
		BC_1_F_6_	5.84-8.5	*K5*	*CIB6*	30.21	47.52	-0.17
		BC_1_F_5_	5.84-8.5	*K5*	*CIB6*	19.44	47.83	-0.21
		BC_1_F_4_	5.84-8.5	*K5*	*CIB6*	38.68	48.68	-0.22
	*QSc.cib-2D.2*	BC_1_F_6_	9.15-11.65	*SSR2650*	*K66*	7.07	9.02	-0.07

Env, environments; LOD, logarithm of odds score; Add, Additive effect (positive values indicate that alleles from Chuanyu12D7 are increasing the trait scores and negative values indicate that alleles from Chuanyu12D7 are decreasing the trait scores); PVE, percentage of the phenotypic variance explained by individual QTL; QTL shown in bold are stable.

The corresponding physical positions of *QSl/Sc.cib-2D.*1 were at 26.11-26.82 Mb on 2DS of the IWGSC RefSeq v2.1. The effects of *QSl/Sc.cib-2D.1* on SL and SC were further analyzed by the flanking markers. As expected, significant differences (*P* < 0.001) on SL and SC were detected between lines with the positive allele from CY25 and Chuanyu12D7 in continuous four generations (**Figure 4**).

### Fine mapping of *QSl/Sc.cib-2D.1* using BC_4_F_2:3_ population

**Figure 5:**
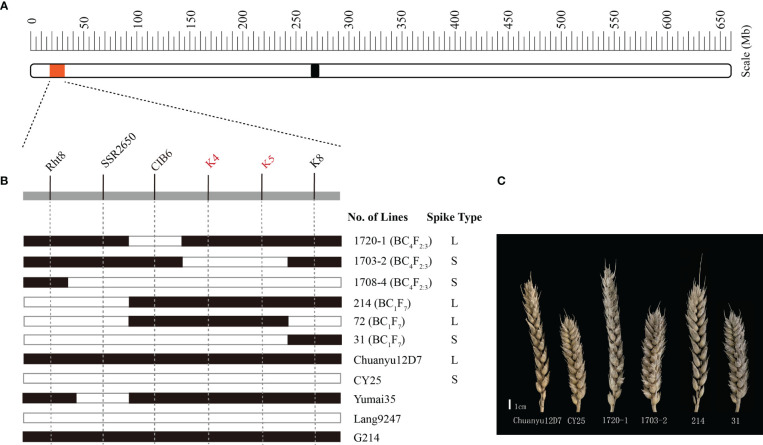
Fine mapping of the major QTL. **(A)** physical location of the region of interest on chromosome 2DS. **(B)** graphical illustration of recombinant genotypes. The filled and open bars represent Chuanyu12D7 type (long spike, L) and CY25 type (short spike, S), respectively. **(C)** The spike of representative lines.

For further verification and fine mapping of *QSl/Sc.cib-2D.1*, all polymorphic markers in target region were used to screen the BC_4_F_2:3_ population (**Supplementary Table 6**). The genetic map was constructed for the BC_4_F_2:3_ population and the order of the markers is the same as in the BILs population. Based on the flanking markers at the target region, several recombinant lines were found derived from the BILs and the BC_4_F_2:3_ populations. The lines with the *K4*-*K5* interval showed an obvious long spike type (L) similar to the parental line Chuanyu12D7, whilst the lines without this interval showed a short spike type (S) similar to the parental line CY25 (**Figure 5**). More specifically, lines such as 1703-2 from the BC_4_F_2:3_ population showed a relatively short spike, whereas 1720-1 showed a long spike. By comparing the genotype and phenotype of different recombinant lines, we concluded that the mapping interval was practical and narrowed down to *K4*-*K5* (**Figure 5B**). Finally, the region of *QSl/Sc.cib-2D.1* was delimited to an interval of 1.71 cM, corresponding to 0.1 Mb between SNP markers *AX-110276364* and *AX-111593853* (**Supplementary Table 6**). Several loci associated with agronomic/morphological traits have been reported near the target region on 2DS, the best known of which is the cloned dwarf gene *Rht8*. After detection of *Rht8* (Chai et al., 2022), it was found that the BILs population was composed of 120 lines with *Rht8* and 144 lines without *Rht8*. The lines with or without *Rht8* both showed significant variation in spike length and spike compactness. Therefore, combining with the fine mapping result, we have proposed that *QSl/Sc.cib-2D.1* is different from *Rht8*, but close to it.

To trace the source of long spike allele of *QSl/Sc.cib-2D.1* in Chuanyu12D7, we genotyped the parents of Chuanyu12D7 with flanking markers. Combining with the pedigree of CY25 (Jinding-1//Yumai35/Lang9247) and Chuanyu12D7 (G214//Yumai35/Lang9247), we inferred that long spike allele of *QSl/Sc.cib-2D.1* inherited from G214 (**Figure 5B**).

### Additive effects of *QSl/Sc.cib-2D.1* and *Rht8* on agronomic traits

**Figure 6:**
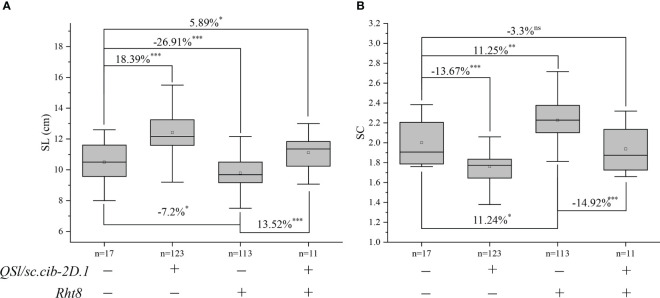
Additive effects of *Rht8* and *QSl/Sc.cib-2D.1* on spike length **(A)** and spike compactness **(B)** in the BIL population. ^*^, ^**^ and ^***^ represented significant level at *P* < 0.05, *P* < 0.01 and *P* < 0.001 respectively. ^ns^ indicates no statistically significant correlation.

Since *QSl/Sc.cib-2D.1* was closely linked with the *Rht8* gene, their coordination effects on plant type and spike type were analysed. Based on the flanking SNP markers *AX-110276364* and *AX-111593853* and the diagnostic marker of *Rht8*, the BILs population was divided into four groups. Compared to lines without *Rht8* and *QSl/Sc.cib-2D.1*, the SL of lines containing only *QSl/Sc.cib-2D.1* increased significantly by 18.39% (*P* < 0.001). On the contrary, the SL of lines containing only *Rht8* decreased by 7.2% (*P* < 0.05). When the positive alleles of *Rht8* and *QSl/Sc.cib-2D.1* were expressed together, SL increased by 5.89% (*P* < 0.05) (**Figure 6A**). On the other hand, the SC of lines with *QSl/Sc.cib-2D.1* presented alone can significantly decrease by 13.67% (*P* < 0.001). The SC of lines containing *Rht8* alone can increase by 11.24% (*P* < 0.05). Lines with a combination of Rht8 and *QSl/Sc.cib-2D.1* showed no statistically significant correlation, compared to lines without either positive allele (**Figure 6B**). It is noteworthy that both on the background of *Rht8*, the lines with *QSl/Sc.cib-2D.1* increased SL by 13.52% and deceased SC by 14.92% compared to the lines without it. The corresponding phenotypic data have been included in **Table 3**. In summary, it has been shown that *QSl/Sc.cib-2D.1* has a strong association with SL and SC. The superior allele of QSl/Sc.cib-2D.1 combined with Rht8 can increase SL and TGW and decrease SC without any apparent trade-offs in other yield-related traits.

**Table 3:** Corresponding phenotypic data.

QSl/Sc.cib-2D.1	Rht8	No. of lines	SL (cm)	SC	PH (cm)	TSS
–	–	17	10.49 ± 1.5	2 ± 0.24	84.31 ± 9.34	21.84 ± 1.62
+	–	123	12.42 ± 1.39	1.76 ± 0.17	88.61 ± 6.95	21.63 ± 1.48
–	+	113	9.79 ± 1.04	2.23 ± 0.2	75.34 ± 5.79	21.63 ± 1.23
+	+	11	11.11 ± 1.12	1.94 ± 0.23	80.16 ± 5.83	21.21 ± 1.61

SL, spike length (SL); SC, Spike compactness; PH, plant height; TSS, total spike number per spike. + represents lines with this gene/locus, while - represents lines without this gene/locus.

**Figure 7:**
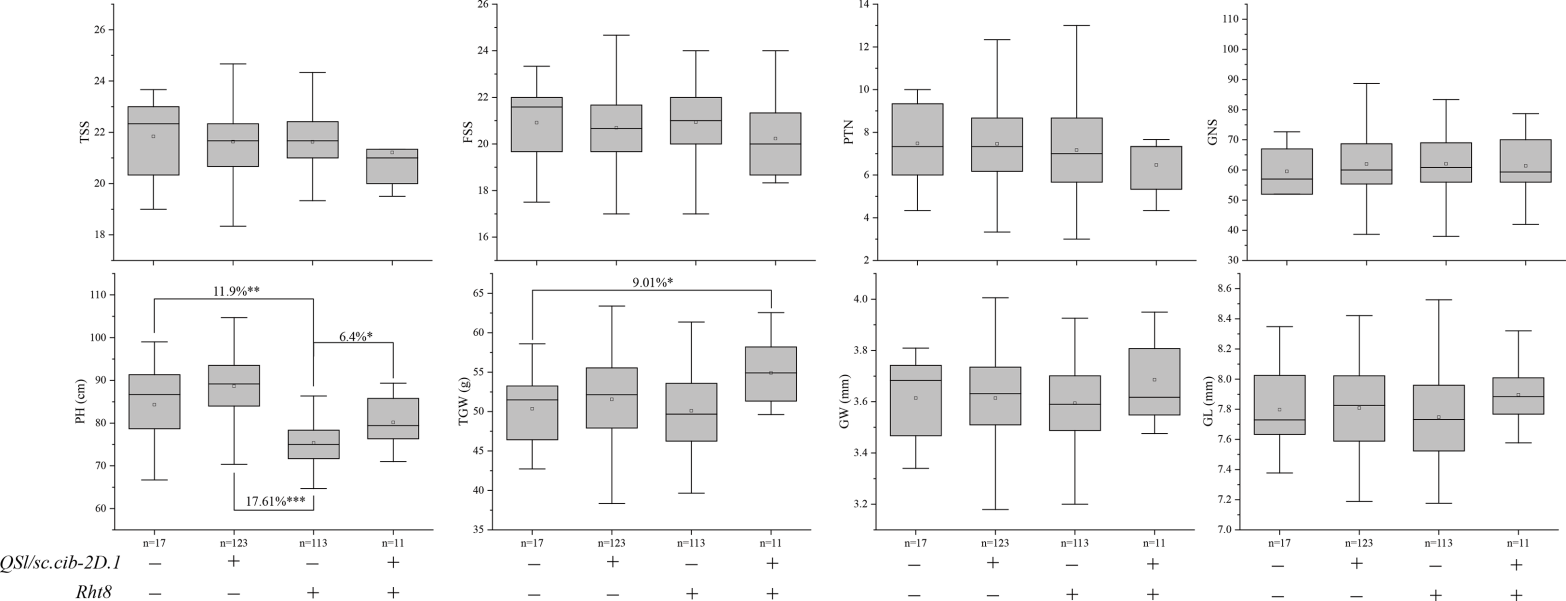
Effects of *Rht8* and *QSl/Sc.cib-2D.1* on other yield-related traits in the BILs population. ^*^, ^**^ and ^***^ represented significant level at *P* < 0.05, *P* < 0.01 and *P* < 0.001 respectively.

We further analyzed the effects of *Rht8* and *QSl/Sc.cib-2D.1* on other yield-related traits (**Figure 7**). Compared to 123 lines possessing positive allele from *QSl/Sc.cib-2D.1*, PH of 113 lines with *Rht8* was extremely and significantly reduced by 17.61% (*P* < 0.001). Compared to lines with or without both positive alleles of *Rht8* and *QSl/Sc.cib-2D.1*, PH of 113 lines with *Rht8* was significantly reduced by 11.9% and 6.4%, respectively. In terms of TGW, lines with a combination of *Rht8* and *QSl/Sc.cib-2D.1* showed a significant increase (~9.01%), compared to lines without either positive allele. Furthermore, no significant differences were found between the four groups of lines for TSS, FSS, PTN, GNS, GW and GL. The above results showed that *QSl/Sc.cib-2D.1* had significant effects on SC, SL, PH and TGW.

### Candidate genes for *QSl/Sc.cib-2D.1*

**Figure 8:**
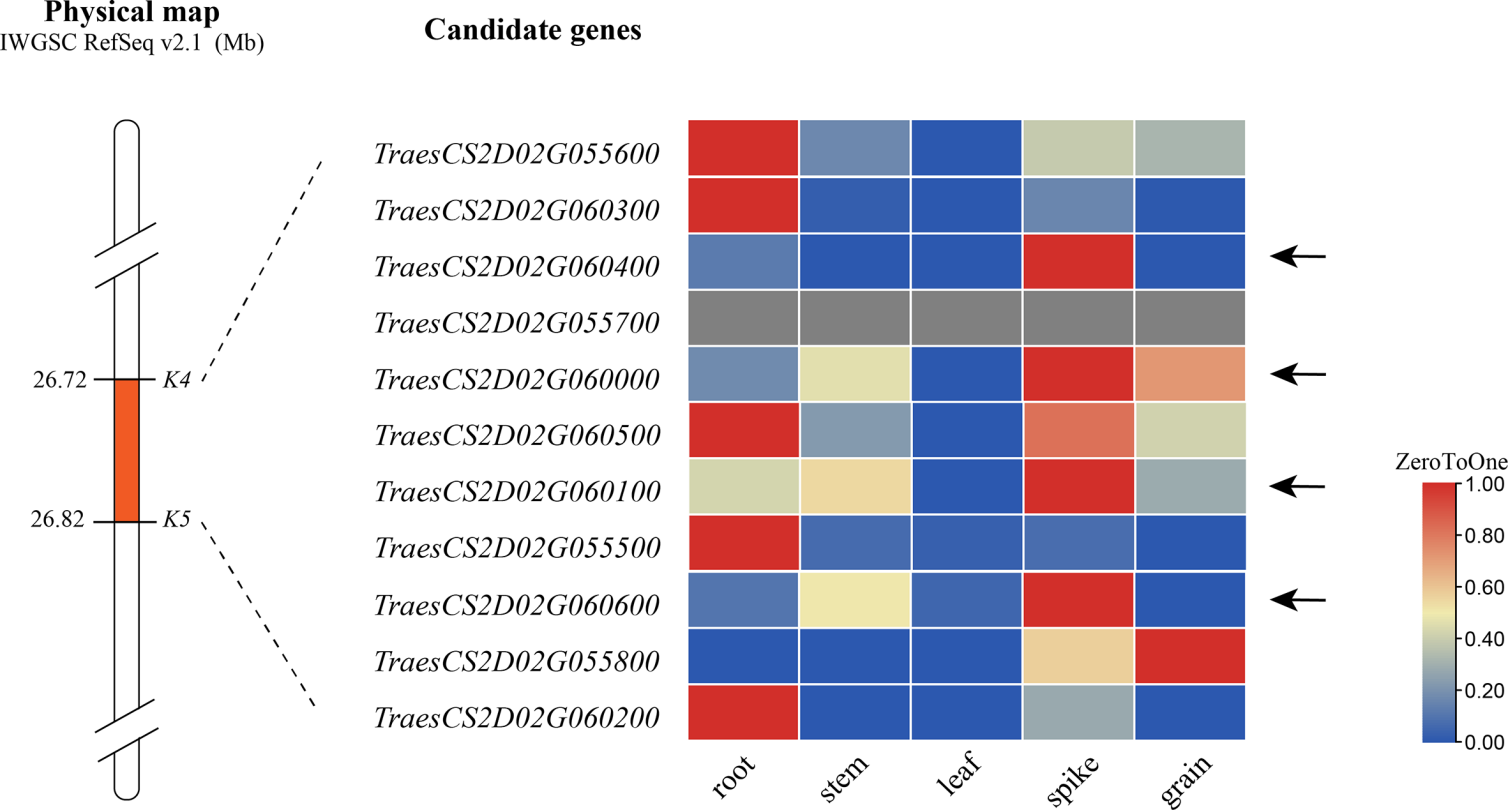
Expression pattern of genes within the *QSl/Sc.cib-2D.1*. The arrows represent candidate genes highly or specifically expressed in spike.

According to the IWGSC RefSeq v2.1, alignment of the flanking markers of *QSl/Sc.cib-2D.1* indicated that it corresponds to a physical interval of 26.72–26.82 Mb on 2DS. There were 11 annotated genes within the candidate intervals. By analyzing the spatiotemporal expression patterns (The International Wheat Genome Sequencing Consortium (IWGSC), 2014; Borrill et al., 2016; Ramírez-González et al., 2018), 4 of 11 genes were found to be highly or specifically expressed in spike (**Figure 8**). These genes are therefore likely to be involved in spike morphology. Among them, *TraesCS2D03G0116500*, *TraesCS2D03G0116600*, *TraesCS2D03G0117100* and *TraesCS2D03G0117500* for *QSl/Sc.cib-2D.1* were probably related to spike length and compactness by combining gene annotation and orthologous gene functions in rice and Arabidopsis (**Supplementary Table 7**). No nonsynonymous SNP was found in the coding region of these five genes based on the 660 K analysis. Further sequencing analysis of these genes is required.

## Discussion

### The application of *Rht* genes and spike morphological QTLs/genes

Wheat breeding has long been focused on improving grain yield and quality. One approach to achieving this goal is through the manipulation of plant architecture and spike development. The *Rht* (reduced height) genes and spike-related genes play important roles in this process. *Rht* genes underpinned the first “Green Revolution” during the late 20^th^ century, thereby reducing lodging and increasing harvest index (Hedden, 2003). This has been facilitated by the identification of more than 20 known *Rht* genes, which are now widely used in global breeding programs.

The *Rht* genes are known to affect plant height by regulating the biosynthesis and sensitivity of gibberellins, a class of plant hormones. Concurrently, the deleterious effects of these genes have been extensively incorporated into commercial cultivars. For instance, the *RhtB1b* (*Rht1*) and *RhtD1b* (*Rht2*) alleles have been shown to result in a reduction in coleoptile length and a decrease in seedling vigor (Li et al., 2011). *Rht4* can increase plant height by 17% and enhance the harvest index while reducing grain weight (Ellis et al., 2005; Gooding et al., 2012). *Rht5* can significantly decrease plant height by 25–55%, but it also reduces grain weight, yield and harvest index (Ellis et al., 2005). However, research has found that its combination with *Ppd-D1* can mitigate its negative effects (Chen et al., 2018). The presence of the *Rht8* gene has been found to result in a decrease in spike length, leading to the formation of a semi-compact spike. However, this genetic modification has also been associated with a reduction in grain and spike number, resulting in a yield penalty (Kowalski et al., 2016). *Rht12* reduces plant height without affecting spike length but decreases grain weight (Rebetzke et al., 2012). *Rht23* (*5Dq′*) gene has been shown to significantly reduce wheat plant height by 40–60%. Additionally, the presence of *5Dq′* has been associated with an increase in spike compactness (Zhao et al., 2018). *Rht25* reduces spike length, increases spike compactness, and decreases TGW (Mo et al., 2018). In addition to *Rht* genes, some plant height QTLs also have pleiotropic effects on spike length and spike compactness (Yu et al., 2020; Liu et al., 2022).

Spike morphology is a multifaceted trait that is influenced by a range of related characteristics including spike length, spike compactness and the number of spikelets. Several spike-related genes/QTLs have been identified that affect these traits. The *Q* gene, located on chromosome 5A of wheat, has been found to confer a free threshing spike and exert pleiotropic effects on plant height and spike length (Liu et al., 2018). The research utilized F_2_ and F_2:3_ lines to identify QTLs associated with traits such as SL, TSS, the number of kernels per spike, and TGW across four distinct environments (Kuang et al., 2020). The findings revealed that wheat yield is directly influenced by its spike traits. Several QTLs were identified for plant height component traits such as total internode number, internode length, and spike length across all environments (Yu et al., 2020). *QSEL.sicau-2CN-5A* has been identified as a novel QTL for spike extension length on chromosome 5AS. This QTL was validated in multiple populations using a newly developed KASP marker that is tightly linked to it. Spike extension length (SEL) is a crucial component of PH and plays an important role in shaping the ideotype of wheat (Li et al., 2020). The application of molecular markers associated with *Rht* and spike-related genes has facilitated the selection of desirable alleles in wheat breeding programs. By bringing together advantageous alleles from multiple loci, breeders can create new varieties with enhanced agricultural performance.

In wheat breeding programs, the application of *Rht* and spike-related genes has led to substantial enhancements in grain yield. However, the potential of spike-related genes to counterbalance the adverse effects of major *Rht* genes has not been thoroughly investigated. In this study, a major QTL, *QSl/Sc.cib-2D.1*, was identified in close proximity to *Rht8*. The combination of *QSl/Sc.cib-2D.1* and *Rht8* was found to increase spike length and thousand-grain weight, while decreasing spike compactness without any apparent trade-offs with other agronomic traits. Further research on the genetic control of plant architecture and spike development will likely lead to more favorable genes/QTLs combinations for crop improvement.

### Comparison of the major QTL with previous studies

In this study, a major QTL *QSl/Sc.cib-2D.1*, comprising *QSl.cib-2D.1* and *QSc.cib-2D.1* loci, was firstly identified in the BIL population. Based on the further validation result, the locus *QSl/Sc.cib-2D.1* at a 1.71-cM interval explained 45.63% and 59.72% of the phenotypic variances for SL/SC and physically located on the chromosome 2D in the interval 26.72-26.82 Mb. The allele originating from Chuanyu12D7 for this QTL contributes positively to a longer and less compact spike. Numerous QTLs for SL or SC were reported on 2DS. To further identify whether this major QTL overlaps with previously reported QTLs, all QTLs were anchored on the CS reference genomes (**Supplementary Table 8**).

It has been proved that *QSl/Sc.cib-2D.1* is a locus controlling SL and SC, which is different from *Rht8*. There were numerous QTLs inferred to be *Rht8* before it was cloned by two groups(Chai et al., 2022; Xiong et al., 2022), such as *QGt/QFt/QRch* (Jantasuriyarat et al., 2004), *QSpl.nau-2D* (Ma et al., 2007), a QTL for SL and SC (Heidari et al., 2011), *QSL-2D/QScn-2D/QPh-2D* (Xu et al., 2014), QSpl.nau-2D (HL1) (Wu et al., 2014), QSl.sdau-2D-1 (Deng et al., 2017), *QSl.cau-2D.2/QSc.cau-2D.2*/*QPht.cau-2D.1* (Zhai et al., 2016) and *QPht/Sl.cau-2D.2* (Chai et al., 2019). Among them, *QSl.cau-2D.2/QSc.cau-2D.2/QPht.cau-2D.1* and *QPht/Sl.cau-2D.2* shared two common parental lines (Jing 411 and Yumai 8679). With an interval comprised *Xgwm261*, the *QSc.cau-2D.1* locus (Zhai et al., 2016) was identical to *QPht/Sl.cau-2D.1* (Chai et al., 2019) which was tightly linked with *Rht8*.

Additionally, *QSl.wa-2DS.e1* was detected in only one environment and located between markers *Xwmc112*–*Xwmc503* corresponding to a physical position of 20.43–24.73 Mb using a temporary F3 population (Wang et al., 2011). For marker-assisted selection (MAS) in breeding programs, only major and stable QTLs have potential value to be used. Therefore, this locus *QSl.wa-2DS.e1* needs further investigation. *QSl.sdau-2D-1* at a 0.7-cM interval between *Xwmc112* and *Xcfd53*, while QSl.sdau-2D-2 at the interval 1.6–47.8 cM was located between SSR markers *Xcfd53*–*Xwmc18* (Deng et al., 2017). *QSl.sdau-2D-1* was anchored to the physical interval 24.73–24.73 Mb and its closely linked flanking marker *Xcfd53* was 9.09 cM away from QSl/Sc.cib-2D.1 in the present study. *QSl.cd-2D.1* controlling spike length at a 34.6-cM interval between *wmc112*–*xgwm484* (Chen et al., 2017). *QSl.his-2D1-1* was identified on 2D in the interval 13.25–36.89 Mb (Xu et al., 2022b), which remains a paucity of evidence on whether it is close to or identical with *Rht8*.

In summary, these studies indicate that the genetic region containing *Rht8* on 2DS has some important QTLs controlling spike-related traits. However, most previous studies have failed to reduce the genetic distance of the QTLs and to determine whether it is identical to *Rht8* using appropriate segregating populations. In the present study, a major and stable locus *QSl/Sc.cib-2D.1* was identified and its correlations with *Rht8* were elucidated using a BC_1_F_7_ and a BC_4_F_2:3_ population. For ideotype breeding, we have successfully broken the linkage between *Rht8* and short spike trait, which can increase spike length while appropriately reducing plant height.

### Coordination effects of *QSl/Sc.cib-2D.1* and *Rht8* for plant type improvement

An ideotype in wheat has a greater potential for yield improvement given its advantages of appropriate assembly of spike morphological QTLs/genes and *Rht* genes. Mining and pyramiding favorable alleles have long been a major goal of molecular breeding. Assembling a group of alleles (i.e., a haplotype block) into a single and optimized genome to generate optimal phenotypes was called genomic design breeding (Xiao et al., 2022), which might be a feasible and efficient strategy to develop high-yield wheat cultivars with ideal plant architecture.

Plant height and spike architecture is important for ideotype breeding given its advantages of appropriate assembly of spike morphological QTLs/genes and *Rht* genes. The utilization of *Rht* genes has been widely used, but the exploration and utilization of spike related genes are still lacking. In the present study, unlike *Rht8*, *QSl/Sc.cib-2D.1* significantly increased SL (~18.39%) and decreased SC (~13.67%) without any apparent trade-offs in other yield-related traits. *Rht8* reduce SL and increase SC without changing the total spikelet number per spike (Kowalski et al., 2016). There were significant differences in plant height and spike length between NIL-Rht8 and NIL-rht8, but no other traits were found, such as TGW, GW, GL, TSS, GNS (Chai et al., 2022). These results were consistent with our results. It is notable that neither of them affects the total spikelet number per spike, suggesting that they affect SL by changing the internode length.

Interestingly, considering *Rht8* had a negative effect on TGW in some cases (Kowalski et al., 2016), the lines carrying a combination of *QSl/Sc.cib-2D.1* and *Rht8* showed significantly higher TGW than other combination lines. A semi-dwarf height with a relatively longer spike length may be a favorable plant type for increasing grain yield. The spike, a part of the plant architecture, is not just an organ that contains grain. It also plays an important role in photosynthetic activity and grain filling. An increase in SL can lead to form bigger grains, ultimately leading to an increased grain yield (Protić et al., 2018). A possible explanation is that a longer spike has a higher photosynthetic activity than a shorter one. Increasing spike length may also increase resistance to fusarium head blight in wheat by reducing spike compactness (Buerstmayr et al., 2009). From the analysis of photosynthetic characteristics, under the premise of selecting long spike rice, the length of spikes should be appropriately increased to reduce the spikelet density, so as to improve the grain filling (Biswal et al., 2021). Therefore, the spikelet distribution of long spike can be more uniform and reasonable, which can improve the favorable conditions for grain filling.

Combining the pedigree and genotype analysis, the superior allele of *QSl/Sc.cib-2D.1* was derived from G214 which has high heritability of long spike and has been used to breed several elite wheat varieties over the past few decades. Genetic factors had a greater influence than environmental factors in determining spike length in wheat (Zečević et al., 2023). Therefore, the positive allele *QSl/Sc.cib-2D.1* from Chuanyu12D7 is of great value for optimizing spike architecture in high-yield wheat breeding. The combination of spike morphological QTLs/genes and *Rht* genes is of great significance for plant type. And plant type optimization is an important part of the yield improvement process. The excavation of spike related genes can not only enrich theoretical understanding of wheat spike development, but also provide more convenient choices for wheat breeding.

### Potential candidate genes for *QSl/Sc.cib-2D.1*

There were 11 candidate genes in the physical interval of 26.72-26.82 Mb **Figure 8**). Further expression analysis, gene annotation and orthologous gene analysis indicated that four of them are likely involved in the spike development (**Supplementary Table 8**). *TraesCS2D03G0116500*, expressed highly and specifically in spike, encodes a Sec1/munc18-like protein which was one of the main regulatory proteins of membrane fusion and related to the mechanism of vesicle fusion during cytokinesis (Rathore et al., 2019). It functions mainly by influencing the expansion of the cells. *TraesCS2D03G0117100* encodes cyclin-like F-box domain-containing proteins that have been reported to be involved in floral transition, panicle and seed development, photomorphogenesis and crop yield (Jain et al., 2007; Gao et al., 2022) (Hong et al., 2020). *TraesCS2D03G0117500* encodes a WD40 repeat-like domain-containing protein which was reported to participate in flowering and panicle development heading date in rice (Jiang et al., 2018), grain number and grain yield in maize and rice (Chen et al., 2022). *TraesCS2D03G0116600* encodes a cleavage and polyadenylation specificity factor subunit involved in floral organ morphogenesis in *Arabidopsis thaliana* (Xie et al., 2015). Therefore, these five candidate genes for *QSl/Sc.cib-2D.1* would be the focus of map-based cloning in the future. Nonetheless, further fine mapping and cloning of this locus will be needed.

## Data availability statement

The original contributions presented in the study are included in the article/**Supplementary Material**. Further inquiries can be directed to the corresponding author.

## Author contributions

MW performed experimental procedures, analyzed results and drafted this manuscript. JL and RL participated in fieldwork. YL, DA, YW, and LZ performed manuscript revision. All authors contributed to the article and approved the submitted version.

## Supplementary material

Supplementary Figure 1 PCR products of *Rht8* CAPS marker. Lane1-6: lines in the BILs population

Supplementary Figure 2 Spike morphology at grain filling stage (A) and rachises of (B) Chuanyu12D7 (left) and CY25 (right). Scale bar = 1 cm.

Supplementary Figure 3 The frequency distribution for SL/SC in BC_1_F_4_, BC_1_F_5_ and BC_1_F_6_ populations

Supplementary Figure 4 The frequency distribution for PH in BC_1_F_4_, BC_1_F_5_, BC_1_F_6 and_ BC_1_F_7_ populations
