# Supplementary figures and images for: Identification and validation of a major quantitative trait locus for spike length and compactness in the wheat (Triticum aestivum L.) line Chuanyu12D7
Source: Front Plant Sci. 2023 Jul 4;14:1186183. doi: 10.3389/fpls.2023.1186183 (PMC10353862; doi:10.3389/fpls.2023.1186183)

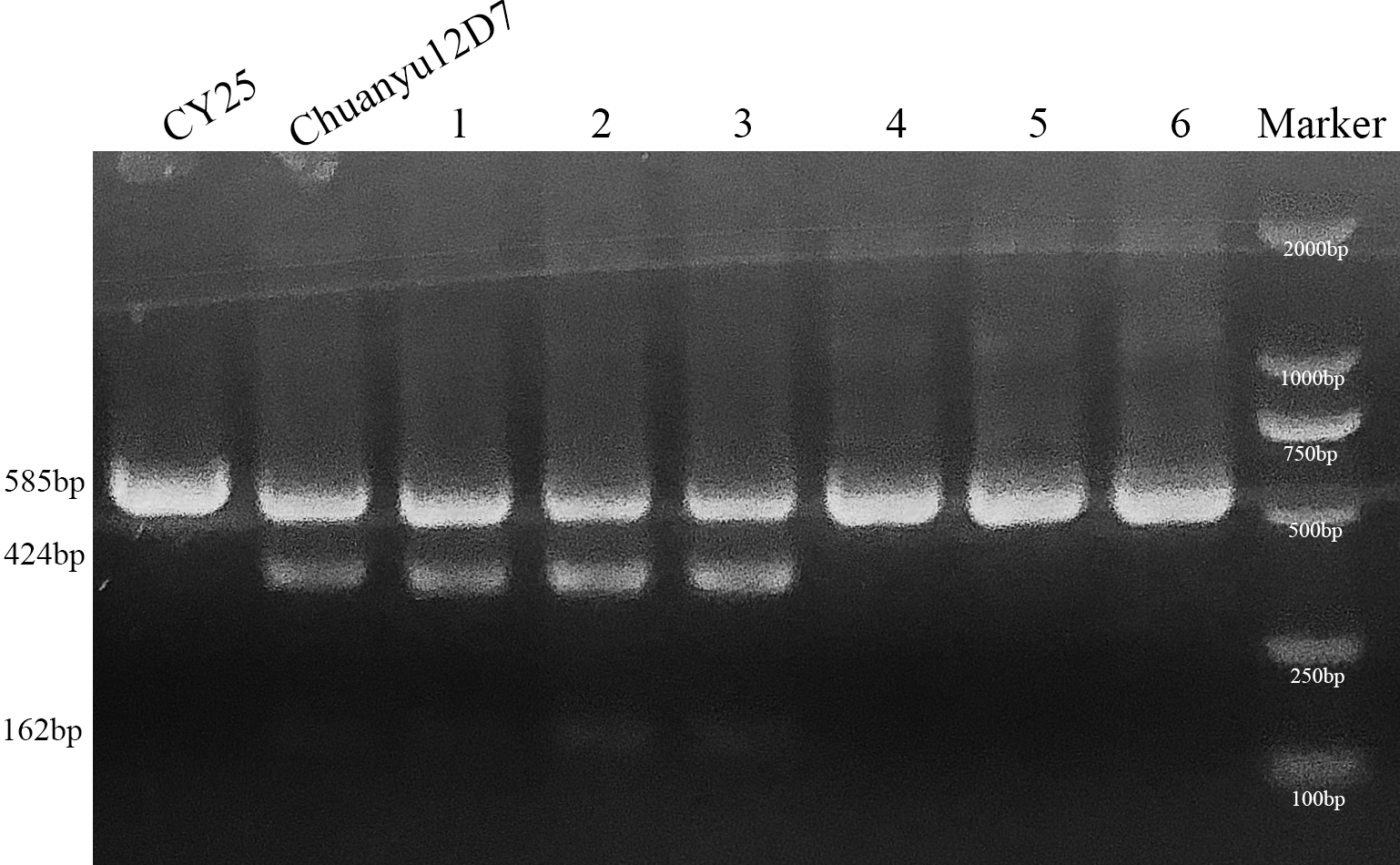

Supplement: Supplementary Figure 1 — PCR products of Rht8 CAPS marker. Lane1-6: lines in the BILs population [file Image_1.tif]

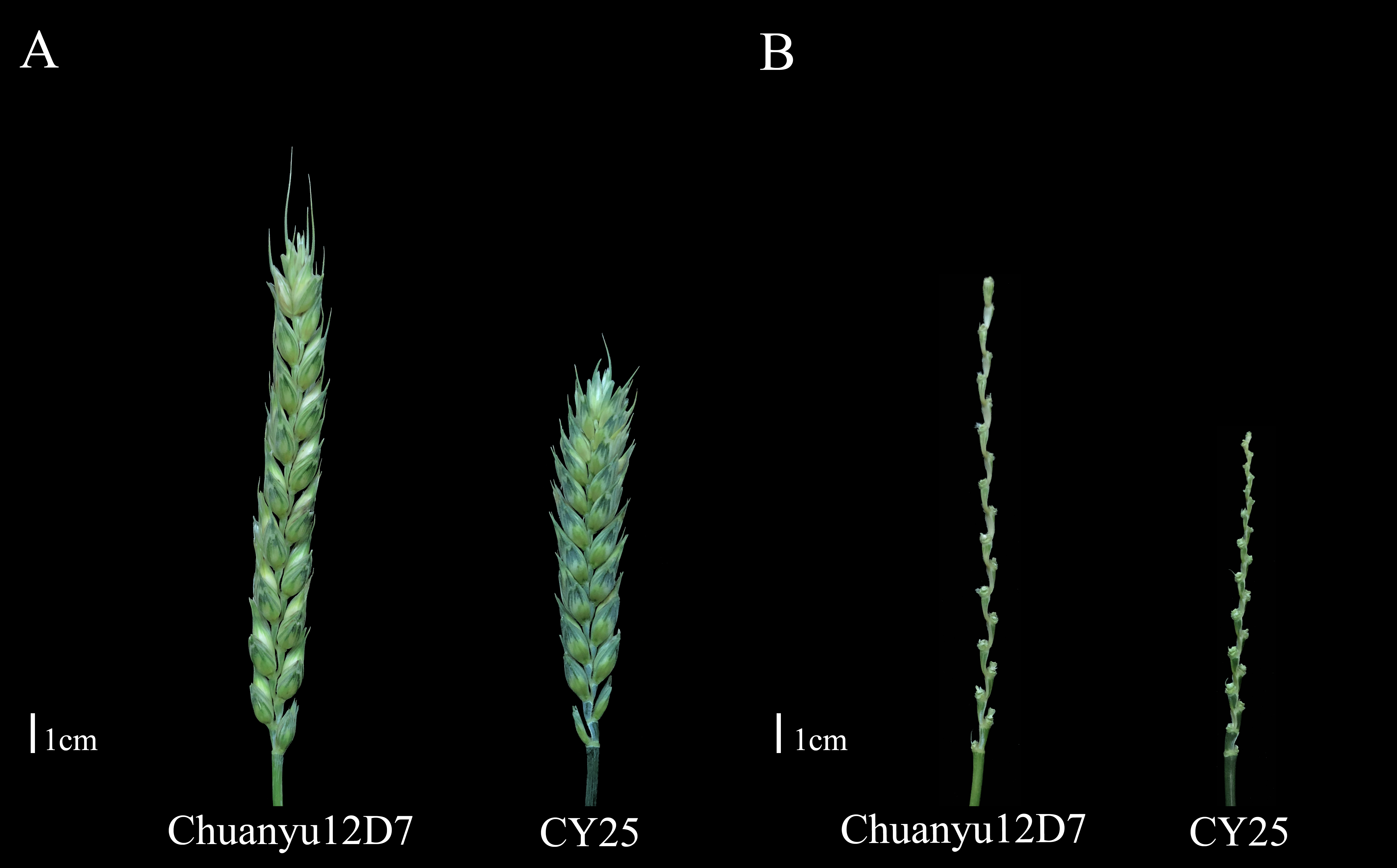

Supplement: Supplementary Figure 2 — Spike morphology at grain filling stage (A) and rachises of (B) Chuanyu12D7 (left) and CY25 (right). Scale bar = 1 cm. [file Image_2.tif]

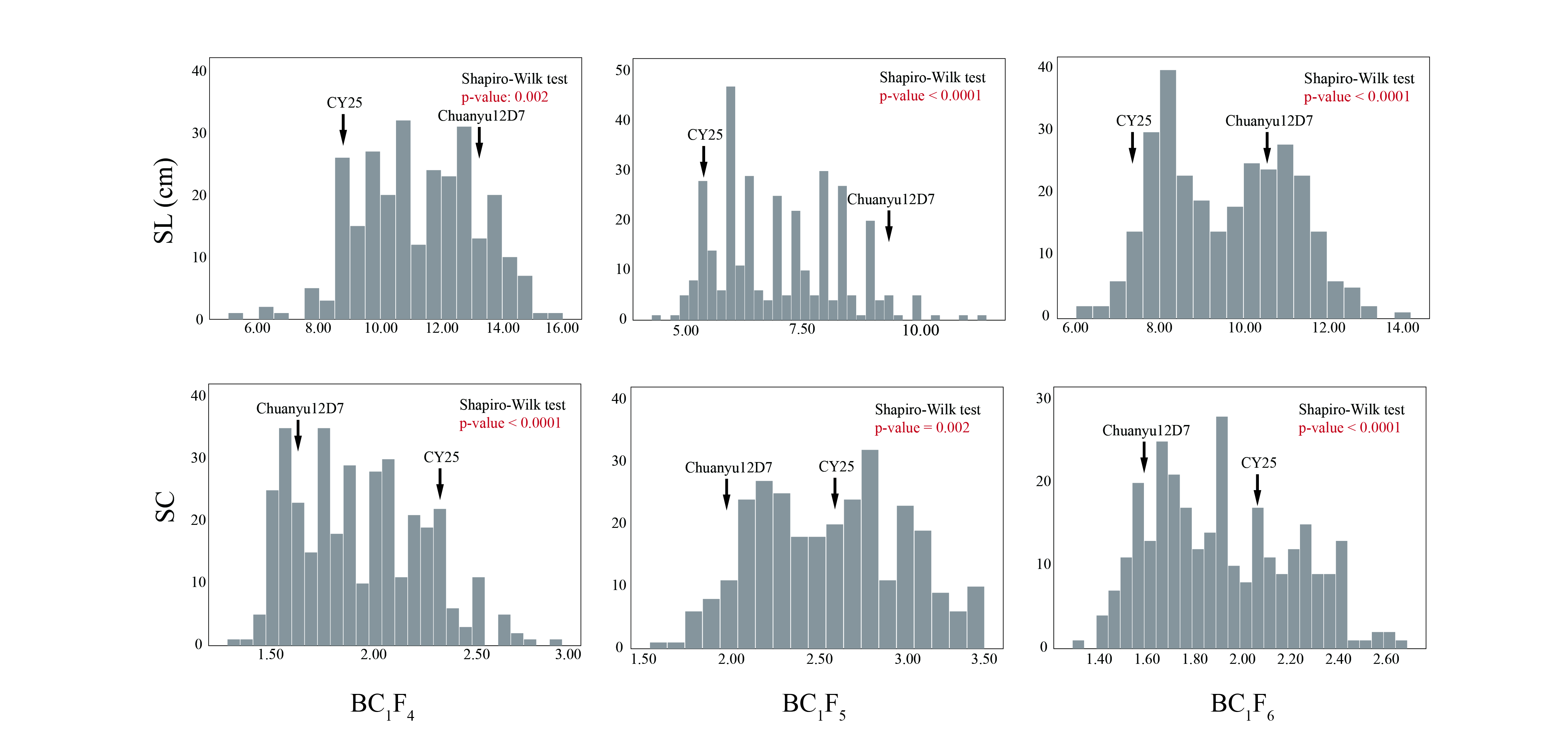

Supplement: Supplementary Figure 3 — The frequency distribution for SL/SC in BC1F4, BC1F5 and BC1F6 populations [file Image_3.tif]

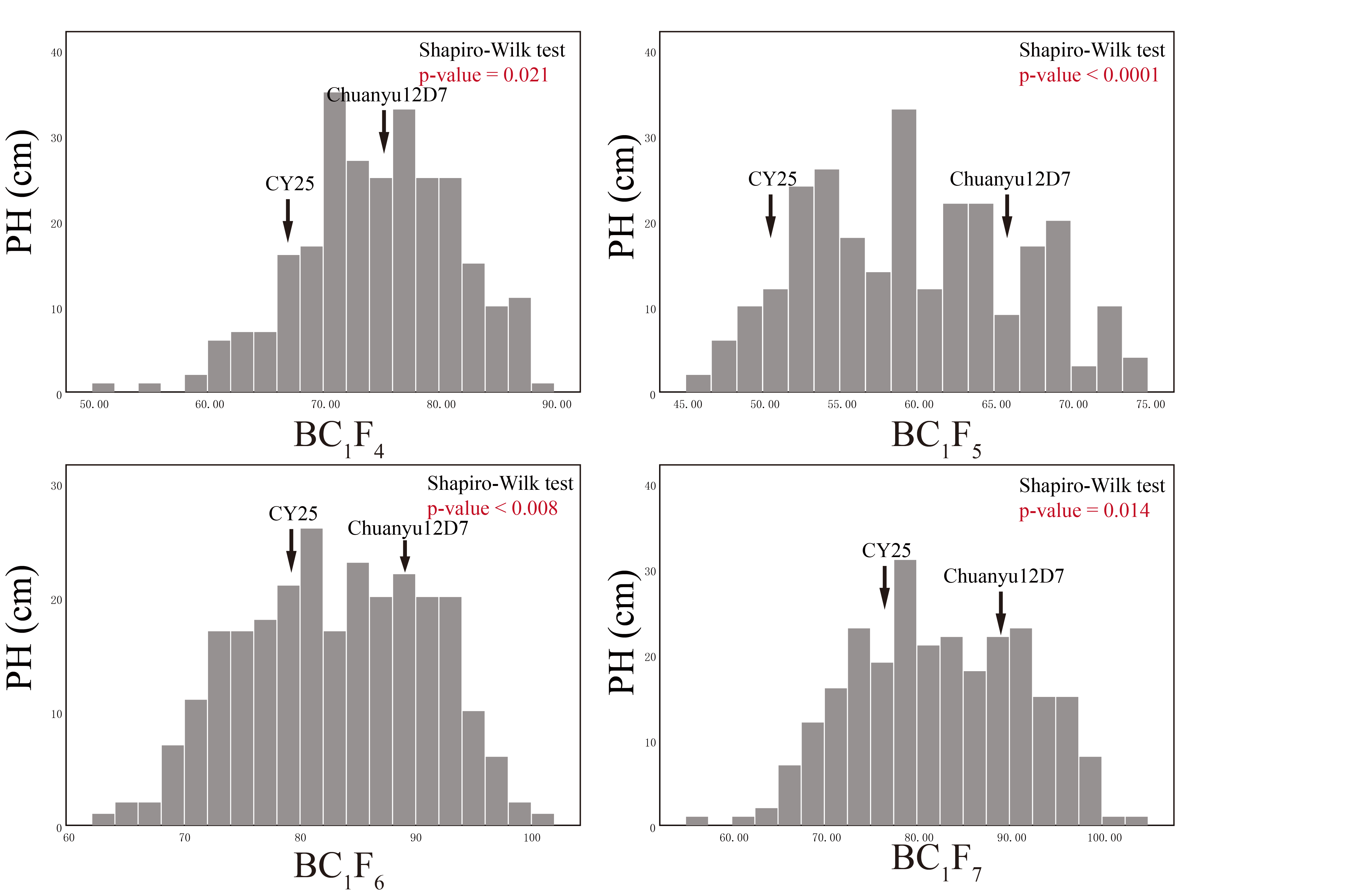

Supplement: Supplementary Figure 4 — The frequency distribution for PH in BC1F4, BC1F5, BC1F6 and BC1F7 populations [file Image_4.tif]
